# Merkel cell carcinoma

**DOI:** 10.1002/ski2.55

**Published:** 2021-06-16

**Authors:** K. Mistry, N. J. Levell, P. Craig, N. M. Steven, Z. C. Venables

**Affiliations:** ^1^ Department of Dermatology Norfolk and Norwich University Hospital Norwich UK; ^2^ Department of Cellular Pathology Gloucestershire Hospitals NHS Foundation Trust Cheltenham UK; ^3^ Institute of Immunology and Immunotherapy University of Birmingham Birmingham UK; ^4^ Public Health England Victoria House Capital Park Cambridge UK

## Abstract

Merkel cell carcinoma (MCC) is a rare neuroendocrine carcinoma. The cellular origin of MCC may include Merkel cell precursors. The incidence of MCC has increased significantly however trends may have been confounded by evolving diagnostic criteria. The two key aetiologies of MCC are ultraviolet radiation and Merkel cell polyoma virus (MCPyV). Both have unique mechanisms of carcinogenesis. MCC presents non‐specifically as a rapidly growing, red‐to‐violet nodule on sun‐exposed areas. Diagnostic accuracy has improved through immunohistochemical markers such as CK‐20. Lymph nodes should be evaluated in MCC through examination and sentinel biopsy. USS, CT, MRI and CT‐PET may be useful in staging. Management depends on tumour location, stage and comorbidities. MCPyV status may guide treatment strategy in the future. Treatment for the primary MCC is commonly wide local excision followed by radiotherapy, guided by anatomical constraints. There is uncertainty about surgical margins. Treatments for nodal disease have not been determined through trials. They include nodal dissection or radiotherapy for clinically or radiologically apparent disease, and adjuvant nodal irradiation for negative nodes, microscopic disease or following nodal dissection for definite disease. Patients with loco‐regional advanced inoperable disease should be considered for combination therapy including chemotherapy, radiotherapy, surgery and immunotherapy. Systemic therapy for advanced disease includes immune checkpoint inhibitors targeting the PD‐1/PD‐L1 pathway. Avelumab can improve survival in metastatic MCC. Immunotherapy may result in longer disease control. Various other immunotherapeutic and molecular agents are undergoing trials. MCC continues to have a high mortality characterized by high recurrence and early metastases.

## Graphical abstract

Merkel cell carcinoma (MCC) is a rare neuroendocrine carcinoma. MCC continues to have a high mortality characterised by high recurrence and early metastases. Diagnostic accuracy has improved through immunohistochemical markers such as CK‐20.

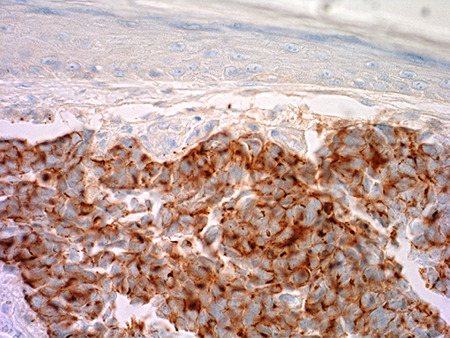

1

**What's already known about this topic?**

- Merkel cell carcinoma (MCC) is a rare neuroendocrine carcinoma. The two key aetiologies are ultraviolet radiation and Merkel cell polyoma virus (MCPyV). Both have unique mechanisms of carcinogenesis.
- MCC presents non‐specifically as a rapidly growing, red‐to‐violet nodule on sun‐exposed areas.
- Treatment for the primary MCC is commonly wide local excision followed by radiotherapy. Patients with loco‐regional advanced inoperable disease should be considered for combination therapy including chemotherapy, radiotherapy, surgery and immunotherapy.

**What does this study add?**

- The incidence of MCC has increased significantly however trends may have been confounded by evolving diagnostic criteria. Diagnostic accuracy has improved through immunohistochemical markers such as CK‐20.
- Management depends on tumour location, stage and comorbidities. MCPyV status may guide treatment strategy in the future.
- Systemic therapy for advanced disease includes immune checkpoint inhibitors targeting the PD‐1/PD‐L1 pathway. Avelumab can improve survival in metastatic MCC.

## 2 INTRODUCTION

This review article presents a concise update of knowledge in these areas relevant to the clinician. This review will discuss the pathogenesis, epidemiology, diagnosis, staging, treatment and prognosis of Merkel cell carcinoma (MCC).

## 3 PATHOGENESIS

MCC is a rare, aggressive neuroendocrine carcinoma first described in Baltimore, USA by Cyril Toker in 1972.^1^ Merkel cells are postmitotic neuroendocrine cells in the basal epidermis which secrete amine and polypeptide hormones. Despite the name, it is unlikely that MCC are derived from Merkel cells themselves. Merkel cells function mainly as touch receptors and the common sites of MCC do not correlate with the common locations of Merkel cells. However, MCC does share much of its immunophenotype and ultrastructure with Merkel cells such that it could be regarded as differentiating towards the Merkel cell phenotype, presumably acquired during carcinogenesis from a precursor cell or cells.^2^ Candidates for the MCC cell of origin include Merkel cell precursors, epithelial progenitors, dermal mesenchymal stem cells, fibroblasts, or even pro‐B or pre‐B cells.^2^

The two main identified contributors to carcinogenesis are ultraviolet radiation (UVR) and Merkel cell polyoma virus (MCPyV). MCPyV is a ubiquitous commensal skin microbiota also carried in other organs and peripheral blood.^3^ It is carried by up to 80% of adults.^3^ A landmark study detected MCPyV in 80% of MCC, although the incidence is lower in other studies perhaps due to an increased proportion of MCC secondary to high UVR exposure.^3^, ^4^ Clonal integration of MCPyV DNA into the genome may drive carcinogenesis. UVR causes immunosuppression and mutagenesis of regulatory genes such as TP53, MYC‐L and RB1.^5^, ^6^ UVR may compound viral carcinogenesis through immunosuppression.^5^

Recent studies have suggested VP‐MCC and VN‐MCC may arise from different cells of origin.^7^, ^8^, ^9^ VP‐MCC from dermal fibroblasts and VN‐MCC from epidermal keratinocytes.^7^ If true, MCC may represent the first malignancy which evolves from cells of origin from two distinct germ layers. Future epigenetic studies may help confirm these distinct lineages. Understanding of the origin, mutational landscape and complex interactions of MCC with the tumour microenvironment has driven the development of targeted immunological and molecular therapies and may give further insight into pathogenesis and therapeutic options.

## 4 EPIDEMIOLOGY

Knowledge of the epidemiology of MCC is improving but older data are lacking in this rare cancer. The Surveillance of Rare Cancers in Europe (RARECARE) database reported an incidence of 0.13 per 100 000 person‐years between 1995 and 2002.^10^ The highest age‐standardized incidence rate globally was reported in Australia between 2012 and 2016 of 2.5 per 100 000.^11^ The highest incidence in Europe was reported in a regional UK study between 2004 and 2013 of 1.78 per 100 000 person‐years.^9^ This was 12‐fold higher than the previously reported UK mean.^12^ By comparison, melanoma was found to be 33‐fold more frequent, but half as fatal as MCC.^13^, ^14^

Controversy remains about actual incidence rates due to evolving diagnostic criteria, improved cancer registration, increased awareness by clinicians, greater availability of diagnostic markers, disease rarity and considerable variation in incidence between developed countries with majority populations of less pigmented skin types.^12^ Since first described in 1972, no epidemiological studies on MCC were reported until 1980.^15^ In 1986, MCC was first allocated a histological code.^15^ From 1992 onwards, cytokeratin‐staining and immunological profiling was increasingly utilized which helped differentiate MCC.^15^ Currently, most cancer registries use the International Classification of Diseases (ICD) 10 coding, which does not have a specific MCC code. MCC is currently coded by ICD‐11, ICD‐oncology‐third edition and some versions of SNOMED. Recent MCC coding expansion may have driven the increased accuracy of epidemiological studies, incorporating data from population‐based registries.^16^ Comparison between countries may be unreliable because different studies use different measures (crude rates or age‐standardized rates with different age standards).^17^ In addition, studies differ in relation to topographic localizations of MCC, for example MCC with unknown primary, that are excluded from their analyses.^17^

Known risk factors include history of UVR, MCPyV, immunosuppression (HIV, transplant, medications and haematological malignancies such as chronic lymphocytic leukaemia), white ethnicity, chronic arsenic exposure, concomitant other malignancies (such as squamous cell carcinoma) and chronic inflammatory disorders.^12^, ^18^ The median age at diagnosis is approximately 76 years and MCC tends to affect men twice more than women although few small cohort studies reported higher incidence in women.^13^, ^19^, ^20^ MCC is roughly 25 times more prevalent in fair‐skinned individuals.^15^ Immunocompromised individuals have earlier onset and higher mortality from MCC.^21^

## 5 DIAGNOSIS

**FIGURE 1:**
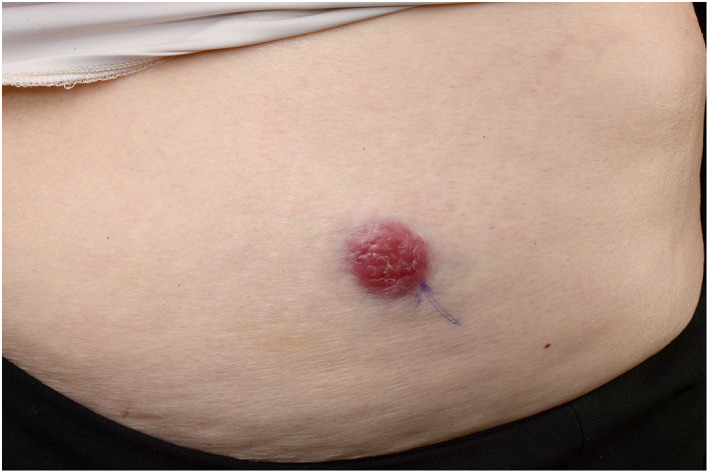
Macroscopic image of a Merkel cell carcinoma showing an erythematous nodule on the right lower abdomen

MCC presents as an irregular, red‐to‐violet cutaneous or subcutaneous nodule (Figure 1).^22^ The important features of MCC can be condensed in the acronym ‘AEIOU’, which stands for asymptomatic/absence of tenderness; expanding rapidly; immunosuppression; older than age 50; UV (exposed site fair‐skinned individuals).^22^ However, alternative presentations have been documented, including cases of the intraepidermal variant of MCC presenting as an erythematous scaly plaque.^9^ The non‐specific presentation of MCC may lead to delayed clinical diagnosis. Common differential diagnoses include amelanotic melanoma, squamous cell carcinoma, cutaneous lymphoma, cutaneous metastasis or benign lesions such as cysts.

**FIGURE 2:**
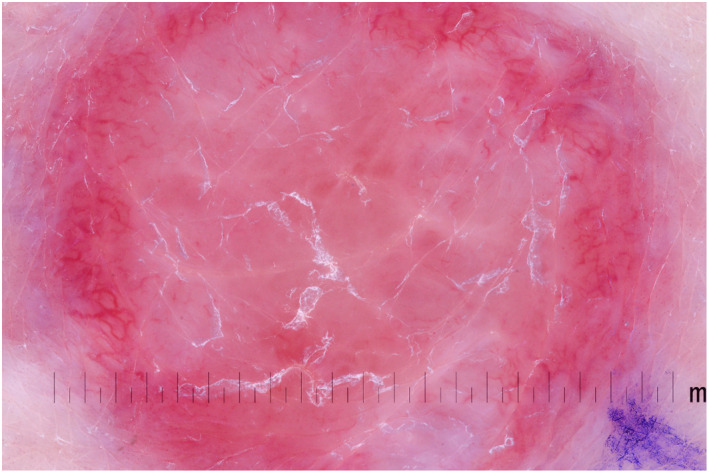
Dermoscopic image of a Merkel cell carcinoma showing a structureless central area with pink and white areas within, peripheral polymorphous and poorly focused vessels are also seen

Due to the wide clinical differential diagnosis, a diagnosis of MCC relies on biopsy and a thorough full body examination of the skin and all lymph nodes. Suggestive features of MCC under dermoscopy include poorly focused vessels, polymorphous vascular pattern with architectural disruption, milky‐red areas on a white sheen, and large calibre arborizing vessels (Figure 2).^23^

**FIGURE 3:**
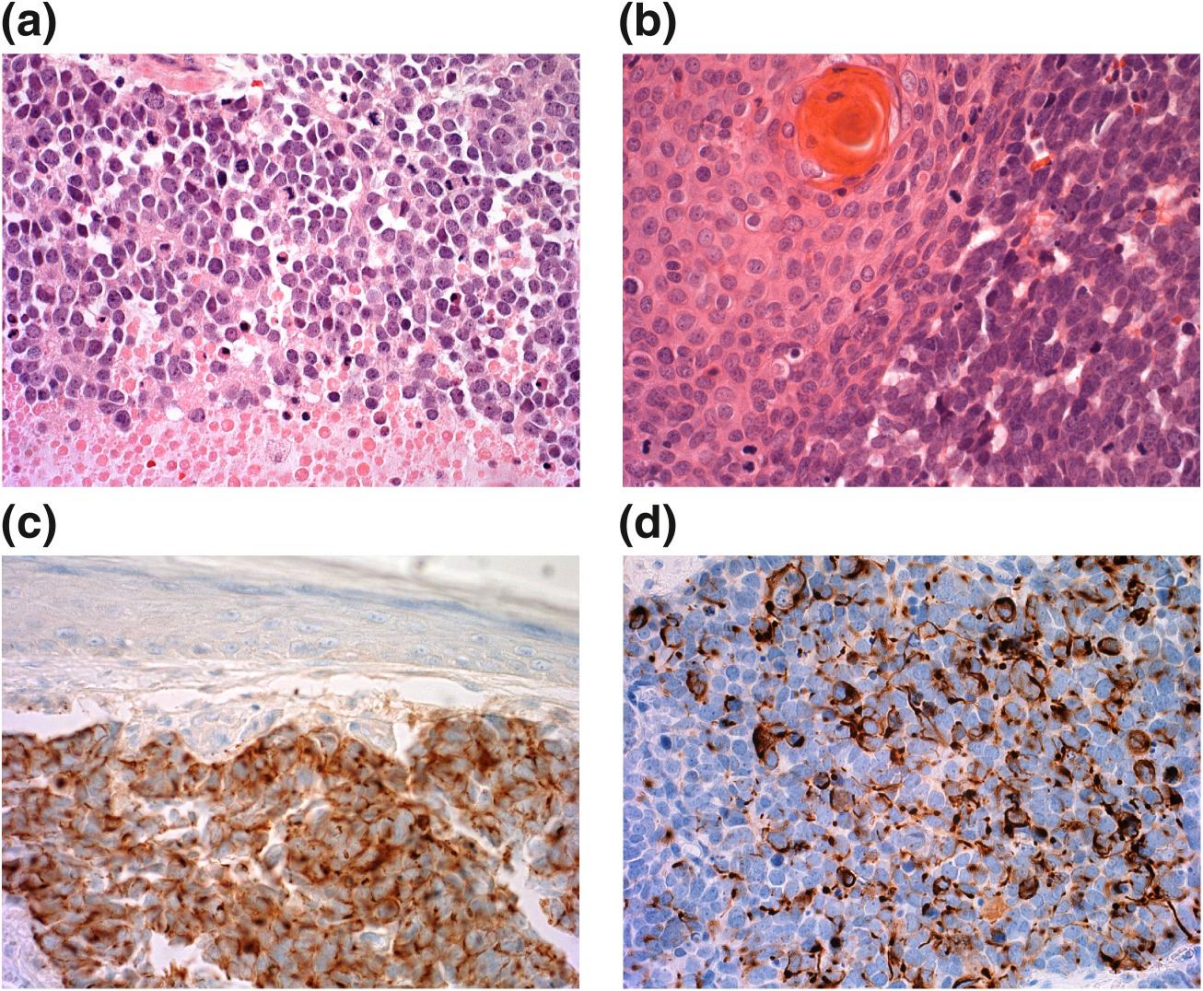
(a) Merkel cell carcinoma (MCC) showing mitotic activity and necrosis of the bottom quarter of the field. H&E ×400. (b) Combined VN‐MCC with MCC component on the left and squamous cell carcinoma component on the right. (c) MCC cytokeratin 20 immunohistochemistry positive (brown cytoplasmic staining) with negative epidermis above. (d) MCC neurofilament protein immunohistochemistry positive (brown cytoplasmic staining)

Tissue sampling is undertaken by either punch or full‐thickness incisional biopsy of the skin. Histological diagnosis requires knowledge of the entity and may be more readily made by specialist dermatopathologists.^24^ MCC stained with haematoxylin & eosin (Figure 3a) reveals a ‘small‐blue‐round‐cell tumour’ composed of dermal and/or subcutaneous nodules or sheets of small, closely packed, monomorphic, round‐to‐oval basaloid cells with a vesicular nucleus, finely granular chromatin pattern and scanty cytoplasm.^25^, ^26^ Numerous mitotic figures and apoptotic/necrotic cells are common. Morphology of the MCC component is very similar in combined VN‐MCC tumours, most commonly in association with a squamous cell carcinoma component (Figure 3b). The morphological differential diagnosis of small blue round cell tumour is wide but can be easily sorted by immunohistochemistry.^26^ Perinuclear staining with cytokeratin 20 (CK‐20) (Figure 3c) has approximately 95% sensitivity and 60% specificity.^27^, ^28^ The identification and mainstream production of CK20 as an MCC marker has improved MCC identification since at least 1997.^29^ Neurofilament protein (Figure 3d) has lower sensitivity but good specificity and is used to detect CK‐20 negative and VN‐MCC, with 96.7% specificity.^28^ Positive immunohistochemistry for MCPyV in VP‐MCC is also a useful diagnostic marker with reliable results using the MCPyV large T‐antigen Antibody (CM2B4) from Santa Cruz Biotechnology (USA).^30^ This mouse monoclonal IgG2b (kappa light chain) is raised against large T/57kT exon 2 peptides of MCPyV; however, it is not widely available in many countries. Pathology reporting of MCC is being standardized internationally including in the 2019 first edition of the International Collaboration on Cancer Reporting (ICCR), MCC Histopathology Reporting Guide.^31^

## 6 STAGING

Staging may aid diagnosis by ruling out other sources of primary neuroendocrine carcinoma, although MCC is by far the most common type to be biopsied in the skin. Cutaneous metastases of other neuroendocrine carcinomas are rare and likely to be end stage with known primary disease elsewhere. Clinically palpable nodes should be assessed by ultrasound and core biopsy or fine needle aspiration.^32^ In those without clinical lymphadenopathy, sentinel lymph node biopsy should be considered because almost one third of patients have occult nodal involvement.^32^ It is unclear whether SLNB confers increased disease‐specific survival with conflicting reports in the literature.^33^, ^34^ CT, MRI and PET‐CT imaging have become increasingly integrated for staging patients who cannot tolerate or decline SLNB, to identify distant metastasis and for surveillance, however imaging cannot substitute SLNB.^35^, ^36^ The most recent staging system is the American Joint Committee on Cancer consensus (AJCC) staging system updated in 2018 (Table 1).^20^, ^37^ However, Union for International Cancer Control (UICC) TNM 8 (Table 2), rather than AJCC TNM 8, has been selected by the Royal College of Pathologists, UK, for pathological staging in the UK because this provides TNM staging of the entire skin surface for cutaneous carcinoma compared with only the head and neck in AJCC 8.^38^ UICC TNM 8 stage's I and II are no longer characterized by SLNB negativity (A) or not done (B), instead the A/B suffix relate to T stage.^38^ Therefore, SLNB has been deprioritised in current staging.

**TABLE 1:** American Joint Committee on Cancer consensus (AJCC) staging system of Merkel cell carcinoma 2018

Stage	Primary tumour	Lymph node	Metastasis
0	In situ (within epidermis only)	No regional lymph node metastasis	No distant metastasis
I Clinical^a^	≤2 cm maximum tumour dimension	Nodes negative by clinical exam (no pathological exam performed)	No distant metastasis
I Pathological^b^	≤2 cm maximum tumour dimension	Nodes negative by pathological exam	No distant metastasis
IIA Clinical	≥2 cm tumour dimension	Nodes negative by clinical exam (no pathological exam performed)	No distant metastasis
IIA Pathological	≥2 cm tumour dimension	Nodes negative by pathological exam	No distant metastasis
IIB Clinical	Primary tumour invades bone, muscle, fascia or cartilage	Nodes negative by clinical exam (no pathological exam performed)	No distant metastasis
IIB Pathological	Primary tumour invades bone, muscle, fascia or cartilage	Nodes negative by pathological exam	No distant metastasis
III Clinical	Any size/depth tumour	Nodes positive by clinical exam (no pathological exam performed)	No distant metastasis
IIIA Pathological	Any size/depth tumour	Nodes positive by pathological exam only (nodal disease not apparent on clinical exam)	No distant metastasis
IIIA Pathological	Not detected (unknown primary)	Nodes positive by clinical exam and confirmed by pathological exam	No distant metastasis
IIIB Pathological	Any size/depth tumour	Nodes positive by clinical exam and confirmed by pathological exam or in‐transit metastasis^c^	No distant metastasis
IV Clinical	Any	± Regional nodal involvement	Distant metastasis detected via clinical examination
IV Pathological	Any	± Regional nodal involvement	Distant metastasis confirmed by pathological exam

^a^ Clinical detection of nodal or metastatic disease may be via inspection, palpation and/or imaging.

^b^ Pathological detection/confirmation of nodal disease may be via sentinel lymph node biopsy, lymphadenectomy or fine needle biopsy and pathological confirmation of metastatic disease may be via biopsy of the suspected metastasis.

^c^ In transit metastasis: a tumour distinct from the primary lesion and located either (1) between the primary lesion and the draining regional lymph node or (2) distal to the primary lesion.

**TABLE 2:** Union for International Cancer Control (UICC) TNM 8

Stage	Primary tumour	Regional lymph nodes	Distant metastasis
Stage 0	Tis	N0	M0
Stage I	T1	N0	M0
Stage IIA	T2, T3	N0	M0
Stage IIB	T4	N0	M0
Stage IIIA	T0	N1b	M0
	T1, T2, T3, T4	N1a(sn), N1a	M0
Stage IIIB	T1, T2, T3, T4	N1b, N2, N3	M0
Stage IV	T0, T1, T2, T3, T4	Any N	M1

TX – Primary tumour cannot be assessed.

T0 – No evidence of primary tumour.

Tis – In situ primary tumour.

T1 – ≤20 mm maximal clinical dimension of tumour.

T2 – >20 mm to ≤50 mm maximal clinical dimension of tumour.

T3 – >50 mm maximal clinical dimension of tumour.

T4 – Primary tumour invades fascia, muscle, bone or cartilage.

NX – Regional nodes cannot be assessed.

N0 – Regional nodes negative by pathological exam.

N1 – Regional nodes positive by pathological exam.

N1a(sn) – Clinically occult but regional node positive by SLNB.

N1a – Clinically occult but regional nodes positive by lymphadenectomy.

N1b – Clinically detected regional nodes.

N2 – In‐transit metastasis without lymph node metastasis.

N3 – In‐transit metastasis with lymph node metastasis.

M0 – No distant metastases.

M1 – Metastasis beyond regional lymph nodes.

M1a – Metastasis to distant skin, subcutaneous tissues or distant lymph nodes confirmed microscopically.

M1b – Metastases to lung confirmed microscopically.

M1c – Metastasis to other visceral sites conformed.

## 7 GENERAL RULES OF TREATMENT

The choice of treatment depends on the stage at presentation, location of the disease, comorbidities and performance status of the patient. Variation in MCPyV incidence in MCC in different countries may lead to different management strategies.^39^ There is no vaccine available against MCPyV and it is unlikely that this will be soon developed given the low incidence of MCC. The interaction between the tumour, the tumour microenvironment and the immune system is likely to be of high importance in response to therapy in this highly immunogenic tumour, stimulating much current research.^40^ MCC management strategy should be established through a multi‐disciplinary approach.^41^, ^42^

## 8 SURGERY

Locoregional disease is commonly treated with wide surgical excision with or without SLNB and adjuvant radiotherapy.^43^ Recurrence following excision ranged from 25% to 40%.^44^ Studies have highlighted a poorer prognosis in larger (>2 cm) primary lesions and in selected small lesions radiotherapy may not be warranted.^45^, ^46^ Clinical excision margins are not proven in trials, with recommended wide local excision margins ranging between 1 and 3 cm down to either muscle fascia or pericranium.^47^ Controversy remains as to the macrographic or micrographic margins required alongside adjuvant radiotherapy. Mohs micrographic surgery may offer reduced local persistence and regional metastasis however no prospective trials have been performed.^48^

## 9 RADIOTHERAPY

Radiotherapy is a treatment option in inoperable patients with primary MCC as monotherapy, with wide‐margin radiotherapy offering in‐field disease control in 75%–100%, however recurrence, disease‐specific and overall mortality are higher compared to operable lesions.^49^, ^50^ Adjuvant radiation to the primary site has become standard of care to reduce local recurrence risk and including the nodal basin if SLNB positive, though trials in this area are lacking.^51^ A retrospective analysis of 4843 patients with localized disease demonstrated improved overall survival with adjuvant radiotherapy compared to surgery alone.^52^ Retrospective studies suggest non‐inferiority of nodal irradiation to complete lymph node dissection (CLND) in SLNB‐positive MCC.^42^, ^53^, ^54^ Randomized trials are required to identify the superior modality. Radiotherapy to the nodal drainage basin may reduce recurrence following CLND.^52^ Radiotherapy may also help reduce pain from bone and other metastasis.^55^ Radiotherapy works by direct dose‐related cytotoxicity.^56^ However, it may also prime T cells augmenting presentation of viral and tumour antigens.^56^ This may explain the poorer outcomes of radiotherapy in immunosuppressed individuals.^57^ Further evidence is required to evaluate the appropriate radiation dose based on patient comorbidities and high competing risk of distant metastases.^58^ Radiotherapy techniques for MCC are discussed further in a recent review.^38^

## 10 CHEMOTHERAPY

MCC is a chemosensitive malignancy with reported overall response rates (ORR) to a range of agents (commonly cisplatin or carboplatin plus etoposide, or anthracycline combinations) up to 75%.^59^ Responses are durable in a minority of patients and survival benefit in the metastatic setting has not been tested in randomized trials compared to no treatment or newer monoclonal antibodies. In particular, it has a role in multi‐modality treatment for challenging MCC loco‐regional disease in which rapidity of response is a priority, enabling downstream surgery and radiotherapy.^60^ A routine role as adjuvant or neo‐adjuvant therapy for operable disease would have to be defined in a randomized trial.

## 11 IMMUNOTHERAPY

There is evidence from uncontrolled trials supporting the use of immune checkpoint inhibitors to treat metastatic or inoperable MCC. The therapeutic target is the programmed death receptor‐1/programmed death ligand‐1 (PD1‐PDL1) immune‐checkpoint pathway that otherwise inhibits effector activity by differentiated T lymphocytes.^61^, ^62^ Both Avelumab (anti‐PDL1 IgG1 monoclonal antibody) and Pembrolizumab (anti‐PD1 IgG4 monoclonal antibody) were licensed by European Medicines Agency (EMA) and Food and Drug Administration (FDA) for metastatic MCC in 2017 and 2018 respectively. Avelumab received UK funding approval in February 2018, as an option for metastatic MCC unresponsive to chemotherapy. In small trials of patients with metastatic MCC naïve to chemotherapy, Pembrolizumab had an ORR of 56% (95% CI 35%–76%) and 6‐month progression free survival (PFS) of 67% (95% CI 49%–86%).^63^ In a similar population, Avelumab, gave an ORR of 62% with >80% ongoing at 6‐months.^64^ In chemotherapy refractory patients, the ORR was lower, 32.8% (95% CI 22%–43%) but with 82% of responses sustained over an average of 10 months.^65^ Though PD‐1/PD‐L1 targeting agents are generally well tolerated, they can trigger a wide range of auto‐immune adverse events, some steroid responsive such as colitis and pneumonitis, and others with life changing consequences, such as optic neuritis and type 1 diabetes.^66^

Intra‐tumoural infiltration by active T lymphocytes and by MCV‐specific T cells is associated with better MCC‐specific survival.^67^ Virus‐negative MCC tumours have a molecular signature characterized by ultraviolet‐induced DNA damage and the presence of tumour‐associated neoantigens.^68^ MCC viral status and mutation burden may influence response to therapy however evidence is required for this to guide treatment choices. Downregulation of MHC class I (MHC I) expression in MCC can be reversed by radiotherapy, IFNβ and chemotherapies, potentially increasing the sensitivity of tumours to subsequent immunotherapy.^69^, ^70^ Radiotherapy may induce the release of tumour‐associated antigens and increase inflammation, thus further synergizing with immunotherapy.^71^ None‐the‐less, routine combination of cytotoxic modalities with immune checkpoint blockade requires evidence from trials.

Approximately 50% of patients with metastatic MCC do not respond or experience disease progression after their initial response to treatment, delineating the need for novel strategies to broaden antitumour immune responses.^72^ Immunosuppressed and elderly individuals are excluded from most clinical trials so the applicability of findings from small trials to real world populations is uncertain. There is evidence suggesting some benefit of re‐exposure to checkpoint inhibitors in a reported series of 13 cases.^73^ Further treatments under investigation include non‐classic immunotherapies such as intratumoural interferon, IL‐12 DNA electroporation, TLR‐4 agonists, adoptive T cell transfer and HLA upregulation.

## 12 MOLECULAR THERAPIES

Treatments targeting mutated or oncogenic driver pathways might have a role in MCC treatment, perhaps immunosuppressed populations. Tyrosine kinase inhibitors and somatostatin analogues have shown promise in small case series but results are yet to be replicated in larger clinical trials.^74^, ^75^

## 13 PROGNOSIS

MCC has a high risk of local‐regional recurrence and early distant metastasis. Five‐year disease‐specific survival ranges from 60% to 87% for those presenting with local disease, 39% to 62% for nodal disease and 11% to 20% for metastatic disease.^17^, ^20^, ^34^, ^76^ Future studies may show that survival has improved since the widespread availability of immunotherapy. MCC usually spreads first to lymph nodes with distant metastases to lungs, adrenal glands, liver and bones.^20^ Three monthly clinical follow up for at least 2 years is recommended. Rarely, spontaneous regression may occur: the mechanism is not understood but this could guide new therapies.^77^ The only reliable prognostic indicator is MCC stage at diagnosis. A large primary tumour and the presence of locoregional or distant metastases are all associated with a poorer prognosis.^20^ Prognostic biomarkers are an area of investigation. Studies of the prognostic role of MCPyV status have, thus far, had mixed results, predominantly finding either a worsened prognosis in patients with VN‐MCC tumours or no difference relative to VP‐MCC tumours.^78^ In VP‐MCC, higher anti‐VP1 and anti‐ST antibodies have been associated with a better prognosis.^79^ Other negative prognostic markers include immunosuppression.^80^

## 14 CONCLUSION

With increasing incidence and high mortality, MCC is likely to be an increasing burden on healthcare resources. However, a lack of international epidemiological data exists for this rare disease. International collaboration and homogeneity in reporting is required to reliably compare epidemiological data. The cellular origin of MCC remains unclear. Identification of the cellular origin may guide management similarly to the discovery of MCPyV and its carcinogenic mechanisms. Clinical diagnosis remains challenging although improvements in cancer registration and immunohistochemistry with increased specialization in cellular pathology and the adoption of a multidisciplinary approach are improving diagnosis, staging and data collection.

Following an International Workshop on MCC research in 2018, the following areas were identified as the highest‐priority research questions^2^: identification of the MCC cell of origin; distinctions between MCPyV positive (VP‐MCC) and MCPyV negative (VN‐MCC) MCC; and the role of these subtypes in guiding management, including combination immunotherapy. Progress in our understanding of MCC tumour immunobiology has led to a rapidly evolving therapeutic landscape and novel immunotherapies have improved prognosis. Five‐year mortality and morbidity are high. Immunotherapy as part of combination therapy may improve outcomes. Monitoring of MCPyV T antigen may prove a useful screening tool for recurrence. The low incidence makes it challenging to power prospective clinical trials. As a result, most management recommendations are based on case series, retrospective reviews, and expert opinion. An international collaborative network is required to establish prospective clinical trials and better establish optimal management.

## CONFLICT OF INTERESTS

No conflict of interests have been declared.

## AUTHOR CONTRIBUTIONS

**K. Mistry:** Conceptualization; Data curation; Formal analysis; Methodology; Visualization; Writing – original draft; Writing – review & editing. **N. J. Levell:** Conceptualization; Supervision; Writing – review & editing. **P. Craig:** Visualization; Writing – original draft; Writing – review & editing. **N. M. Steven:** Validation; Writing – review & editing. **Z. C. Venables:** Conceptualization; Methodology; Supervision; Writing – original draft; Writing – review & editing.

## Data Availability

Data sharing not applicable to this article as no datasets were generated or analysed during the current study.
